# Why do people participate in mass anti-malarial administration? Findings from a qualitative study in Nong District, Savannakhet Province, Lao PDR (Laos)

**DOI:** 10.1186/s12936-017-2158-4

**Published:** 2018-01-09

**Authors:** Bipin Adhikari, Koukeo Phommasone, Palingnaphone Kommarasy, Xayaphone Soundala, Phonesavanh Souvanthong, Tiengkham Pongvongsa, Gisela Henriques, Paul N. Newton, Nicholas J. White, Nicholas P. J. Day, Arjen M. Dondorp, Lorenz von Seidlein, Mayfong Mayxay, Phaik Yeong Cheah, Christopher Pell

**Affiliations:** 1 Mahidol-Oxford Tropical Medicine Research Unit, Faculty of Tropical Medicine, Mahidol University, Bangkok, Thailand; 2 Centre for Tropical Medicine and Global Health, Nuffield Department of Medicine, Churchill Hospital, Oxford, UK; 3 Kellogg College, University of Oxford, Oxford, UK; 4 Lao-Oxford-Mahosot Hospital-Wellcome Trust Research Unit (LOMWRU), Microbiology Laboratory, Vientiane, Laos; 5 Savannakhet Provincial Health Department, Savannakhet, Savannakhet Province Laos; 6 Faculty of Postgraduate Studies, University of Health Sciences, Vientiane, Laos; 7 The Ethox Centre, Nuffield Department of Population Health, University of Oxford, Oxford, UK; 8 Centre for Social Science and Global Health, University of Amsterdam, Amsterdam, The Netherlands; 9 Amsterdam Institute for Global Health and Development, Amsterdam, The Netherlands

**Keywords:** Malaria, Qualitative, Coverage, Factors, Trust, Mass drug administration, Partnership, Volunteers, Laos

## Abstract

**Background:**

As a part of targeted malaria elimination (TME) in the Greater Mekong Sub-region (GMS), mass drug administration (MDA) with anti-malarials was conducted in four villages in Nong District, Savannakhet Province, Lao PDR (Laos). A high proportion of the target population participated in the MDA, with over 87% agreeing to take the anti-malarial. Drawing on qualitative data collected alongside the MDA, this article explores the factors that led to this high population coverage.

**Methods:**

Qualitative data collection methods included observations, which were recorded in field notes, focus group discussions (FGDs), and semi-structured interviews (SSIs). Data were collected on local context, MDA-related knowledge, attitudes and perceptions. FGDs and SSIs were audio-recorded, transcribed and translated to English. All transcriptions and field notes underwent qualitative content analysis using QSR NVivo.

**Results:**

Respondents recognized malaria as a health concern and described the need for a malaria control program. The risk of malaria including asymptomatic infection was explained in terms of participants’ work in forest and fields, and poor hygiene. During the MDA rounds, there was an improvement in knowledge on the concept of asymptomatic malaria, the rationale of MDA and the blood test. In all four villages, poverty affected access to healthcare and the provision of free care by TME was highly appreciated. TME was jointly undertaken by research staff and local volunteers. Authorities were involved in all TME activities. *Lao Theung* communities were cohesive and community members tended to follow each other’s behaviour closely including participation in MDA. Factors such as understanding the concept and rationale of the study, free health care, collaboration with the village volunteers, support from authorities and cohesive communities contributed in building trust and high population coverage in MDA.

**Conclusion:**

Future malaria control programmes can become successful in achieving the high coverage in MDAs drawing from the success of TME in Laos. A high population coverage in TME was a combination of various factors that included the community engagement to promote the concept and rationale of MDA for asymptomatic malaria in addition to their baseline understanding of malaria as a health concern, provision of free primary health care, partnering of the research with local volunteers and authorities, building social relationship with community members and the cohesive nature of the communities boosted the trust and participation in MDA.

**Electronic supplementary material:**

The online version of this article (10.1186/s12936-017-2158-4) contains supplementary material, which is available to authorized users.

## Background

The emergence of multi-drug resistant *Plasmodium falciparum* in western Cambodia is a serious threat to current malaria control efforts [1–5]. In response, a range of activities have been undertaken to contain its potential spread to westwards [6, 7].

Targeted malaria elimination (TME), a package of interventions, which includes mass anti-malarial administration and the strengthening of village malaria worker networks, has been recently piloted in several communities across the GMS [6]. As part of TME, dihydroartemesinin piperaquine (DHA/PQ) and single, low-dose primaquine are offered to all members of a target community every month for 3 months. This is followed by a year of quarterly parasitaemia surveys by uPCR (ultrasensitive polymerase chain reaction) [6]. To evaluate the effectiveness of TME in terms of interrupting local malaria transmission, communities with reservoirs of asymptomatic malaria infections have been targeted on the Thai-Myanmar border [8, 9], Myanmar [10], Cambodia [11, 12], Vietnam [13] and Laos [14].

The potential of interrupting *P. falciparum* transmission through mass anti-malarial administration depends on local malaria transmission dynamics, effectiveness of the anti-malarial and coverage in the target population [15–17]. To promote the uptake in target communities, a range of activities, including health education through theatre, posters, village meetings and house-to-house visits, have been undertaken alongside mass anti-malarial administration [15]. The effectiveness of these “community engagement” activities—in terms of increasing coverage—is intertwined with the local social and cultural context [10, 11].

To date, social scientists have used qualitative methods in Myanmar [10] and Cambodia [11] to explore how local social circumstances and community engagement activities influence coverage of mass anti-malarial administration within TME. In view of potential mass-antimalarial administration campaigns across the region, further research is needed to examine the community responses to TME in target areas of the GMS that are socially and culturally distinct. This article examines how the community engagement activities and the local social and cultural context affected the uptake of mass-antimalarial administration in target villages in Laos.

## Methods

### The local social context

**Fig. 1:**
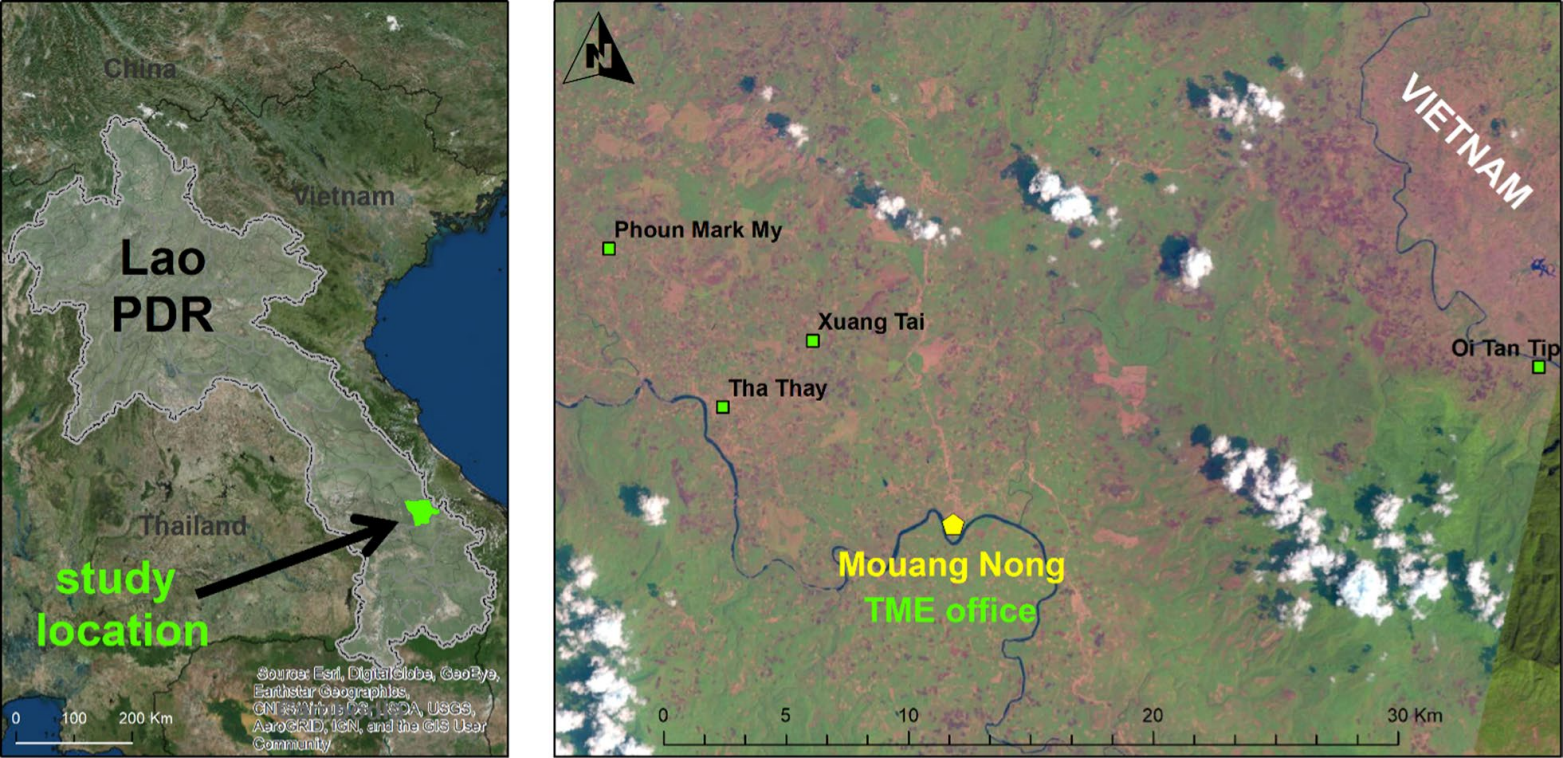
TME study sites in Savannakhet Province, Laos

The four TME villages are located in Nong District, one of the poorest districts in Laos [18, 19] (Fig. 1). The local population is comprised of members of the *Lao Theung* ethnic minority, who are Mon-Khamer-speaking aboriginals. Villages comprised population between 300 and 500 residents: Oi Tantip (OTP = 512), Phoun Mak Mee (PMM = 480), Thate (TT = 526) and Xuang Tai (XT = 371) [20]. Physically isolated and lacking transportation, community members have limited access to education, health and the district headquarters.

The villages are located amongst hills and forest and residents’ livelihoods depend upon the migratory swidden cultivation. Almost all residents also rear livestock, such as cows, chicken, pigs and goats as a source of emergency cash. Among the *Lao Theung*, culture, familial integrity and conformism within households are highly valued attributes. Household heads or senior family members therefore are often influential in relatives’ decisions [21, 22]. Within villages, a tendency for communal decision-making, sharing labour and helping one another, is rooted in their ethnic identity [21]. Village decisions are often made at the community meetings led by the village head and the other leaders, such as senior, security head, village volunteers, health volunteers and women union head [20].

Within the TME villages, healthcare facilities are inadequate: one village (PMM) has a health centre; the distance to the nearest health centre in TT is around 5 km; XT residents’ nearest facility is about 7 km away; and for residents of OTP the nearest health centre is about 10 km away from the village (Fig. 1). The catchment population for a health facility varies across the villages. In general, one health center was responsible for three to five villages (1500–2500 residents). The facilities are often under-resourced in terms of the continuous presence of health workers, a lack medical supplies and the services they could offer (each center consisted of three to four staff headed by a medical assistant). Based on the complaints that TME health staff were asked to treat during the study, a large proportion of the health problems in the villages were infectious diseases, such as febrile illnesses, diarrhoea, respiratory illnesses, conditions related to hygiene (e.g. skin infections) and maternal and child health conditions.

### Targeted malaria elimination

**Fig. 2:**
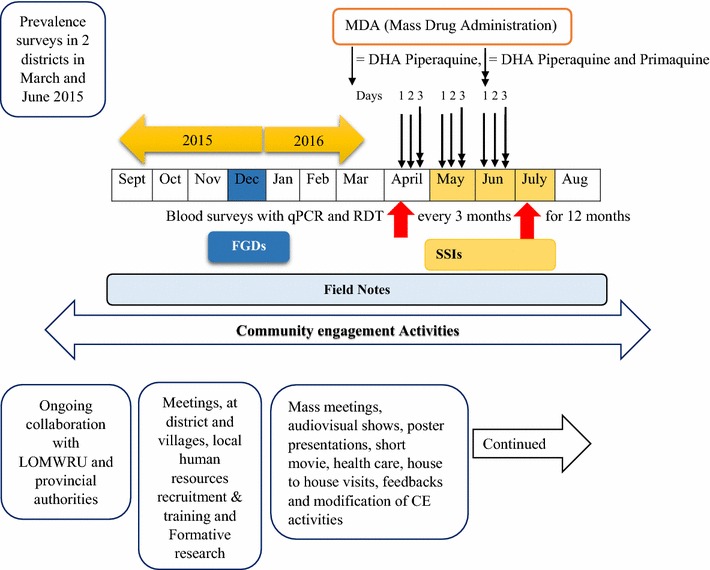
Schematic representation of various data collections alongside TME and community engagement

The effectiveness of TME was evaluated through a cross-over randomized controlled trial in four villages (OTP, PMM, TT and XT) in Nong District (Figs. 1, 2). Target villages were selected based on the prevalence of *P. falciparum* established beforehand [14]. Among these four villages, two were randomized as intervention villages (PMM and TT) and two as control villages (OTP and XT). Intervention villages received MDA of dihydroartemesinin-piperaquine (DHA-PPQ) for 3 days and a single low dose primaquine every month for 3 months, followed by blood surveys every 3 months for 1 year. Blood surveys in intervention and control villages involved collection of venous blood sample (3 ml from adults and 0.5 ml from children ≤ 5 years) from all participants. Based on previous research, the target population coverage for TME was 80% [15, 16]. Among total population of 1017 in two intervention villages, 973 were eligible for MDA, after excluding infants under 6 months, pregnant women and severely sick people. In the two intervention villages, 855 of 973 (88%) of residents participated in the three rounds of drug administrations (nine doses DHA-PPQ and three doses PQ) [20, 22]. Control villages underwent quarterly blood surveys by qPCR for 12 months, but received the same MDA at the end of the 12-month surveillance period [20].

### Community engagement

**Fig. 3:**
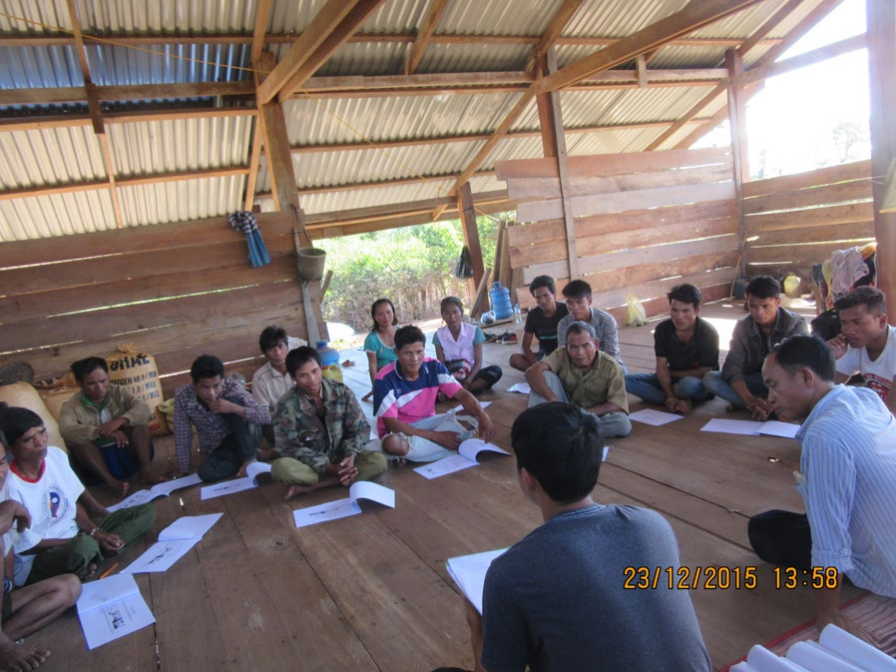
Volunteers training using the malaria guide book and posters at XT

**Fig. 4:**
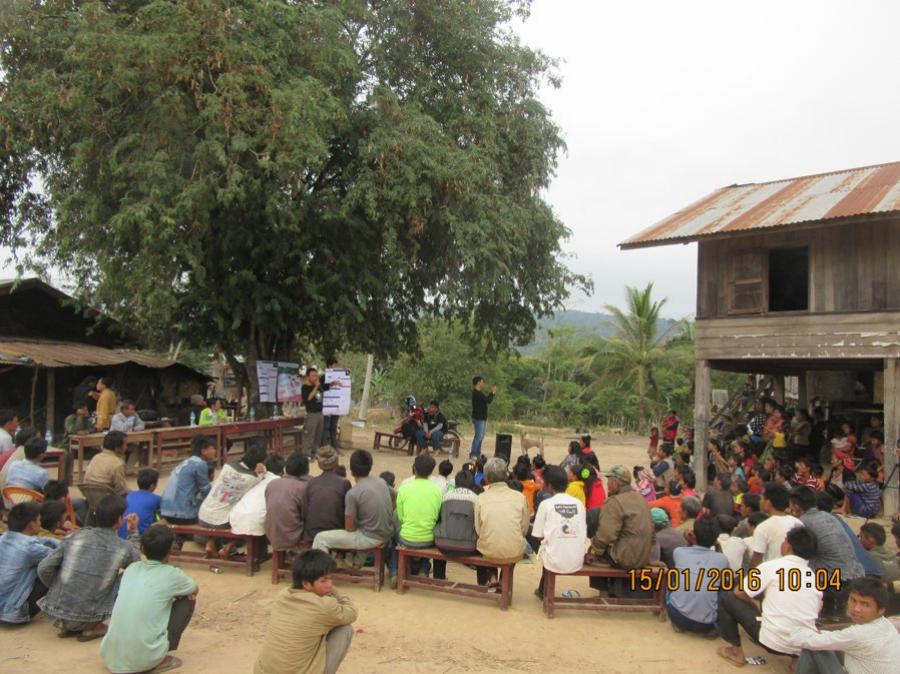
A mass meeting in TT, explaining TME procedures using a poster

As part of TME, community engagement activities were undertaken to promote the uptake of MDA and blood surveys. Drawing on past experience, a systematic literature review [15], formative research and ongoing feedback from stakeholders, community engagement was tailored to the local context [20] (Additional file 1). This entailed a stepwise process of collaborating with the authorities at province, district and the villages. In the villages, volunteers (10/each village) were selected by TME team and the village head, trained and—together with the TME team—helped design and implement community engagement [20]. This included the design of educational tools, such as audio-visual shows, a malaria guidebook, posters, and locally made videos which consisted the messages on the concept of asymptomatic malaria, rationale of MDA and blood test and were used during community meetings and home visits (Additional files 1, 2; Figs. 3, 4) [20]. Community engagement activities were similar across all four (intervention and control villages) villages. However, as the residents of intervention villages were offered anti-malarials in the first year, community engagement, including health care was tailored and intensified in response to the reported adverse events and rumors [20].

Participants were reimbursed, 100,000 Kip/for 3 days (~ USD 12), for their travel and opportunity costs when participating in TME activities, such as blood surveys or MDA. As a token of appreciation for hosting the study, all participants were provided cooking utensils (a set containing bowls, plates and spoons), T-shirts printed with the malaria prevention message and blankets during the cold season. As a part of malaria control efforts, long lasting insecticidal nets (LLINs) were provided to all community members. During mass meetings, blood surveys and MDA rounds, participants were offered snacks (e.g. soya milk, cake, toffies and biscuits).

All residents were provided with the free primary health care, additional financial support (transport and hospital cost) for any severe cases (in addition and beyond the study disease: malaria or adverse events due to anti-malarials) when they were referred to the higher health centers [20]. In each village and sub-village, the need for potable water was met by installing the water pumps (n = 8) during the study period [23].

Community engagement also incorporates the wider aim of engendering ethical global health research through, for example, socially responsible knowledge production, capacity building and health promotion, through sustainable changes in health behaviour [15, 24–26]. Although community engagement in TME largely aimed at promoting population coverage, some of the sustainable effects of community engagement, for example, inherent in health education and health promotion were noteworthy [22]. Health education about malaria, the benefits of early health-seeking behaviour, preventive behaviours, such as using bed nets and the knowledge on risk of malaria may have increased the health (malaria) literacy [20, 22]. In the villages, local village volunteers were also trained to diagnose a suspected case of malaria using RDT and provide an anti-malarial under the supervision of TME physician and/or health centre staff [20]. Overall cleanliness and hygiene was often discussed with community members during meetings and potable water pumps were installed in the villages. These components may well have a longer term benefit to the communities. Nevertheless, because TME was a clinical trial, the accompanying activities, including health care could not be continued after the project ended in June, 2017.

### Data collection

Data were collected alongside the main TME study from September 2015 to August 2016. Data collection included observations of study and community engagement activities (recorded in field notes), focus group discussions (FGDs), and semi-structured interviews (SSIs).

### Focus group discussions

A total of 12 FGDs, six each in intervention (two in PMM and four in TT) and control villages (four in OTP and two in XT) that included additional FGDs for the sub-villages, were conducted in December 2015 (Fig. 2 and Table 1). Focus group discussions were conducted as a part of formative research before the TME started to inform and design the community engagement: the aim was to explore the socio-demographic status, health-seeking behaviour, knowledge, attitudes and perceptions towards malaria and MDA. To select participants, the village head was asked to invite all interested participants to a village meeting. Eight to ten community members were randomly chosen for each of the FGD. A female social scientist conducted FGDs with female participants at one of the participant’s house and a male social scientist conducted FGDs with male participants. All FGDs were conducted in *Lao Theung* and were translated on site by a fluent bilingual local volunteer or a local coordinator, who were trained on the topics covered in FGDs. Topics addressed in the FGDs included malaria (including the concept of asymptomatic infections by malaria parasites) and the rationale of MDA. The FGDs were audio-recorded after informed written consent was sought from each participant. Additional note taking was carried out by an assistant to record non-verbal reactions, expressions and general atmosphere of the discussion.

**Table 1:** Socio-demographic characteristics of FGD participants (n = 100)

Characteristics	Total	Villages	
		Intervention (n=52)	Control (n=48)
	Number (%)	Number (%)	Number (%)
Age group (years)			
18–30	38 (38)	19 (36.5)	19 (39.6)
31–40	32 (32)	20 (38.5)	12 (25)
≥ 41	30 (30)	13 (25)	17 (35.4)
Mean = 37.58 ± 12.71; range=18–80 years			
Sex			
Female	48 (48)	24 (46.2)	24 (50)
Male	52 (52)	28 (53.8)	24 (50)
Occupation			
Farmer	100 (100)	52 (100)	48 (100)
Education groups			
Not attended school	82 (82)	44 (84.6)	38 (79.2)
1−5 years	16 (16)	7 (13.5)	9 (18.8)
>6 years	2 (2)	1 (1.9)	1 (2.1)
Name of the villages			
Oi^a^	16 (16)	0	16 (33.3)
Tantip^a^	16 (16)	0	16 (33.3)
Phounmakmee	18 (18)	18 (34.6)	0
Keng and Appok	18 (18)	18 (34.6)	0
Thate Main	16 (16)	16 (30.8)	0
Xuang Tai^a^	16 (16)	0	16 (33.3)

^a^Control villages

### Observations and field notes

Field notes were collected based on the observation of study-related activities, such as meetings, volunteer’s training, audio-visual shows, mass meetings, house-to-house visits and MDA. Field notes contained the location, subject of the notes, date and discussions during meetings including the reflection by the observer/note taker. Field notes were made by BA, XS and PK in English with on-site translation where necessary. A total of 130 field notes were collected from September 2015 to August 2016. Field notes included descriptions of the activities and summaries of informal conversations about the study.

### Semi-structured interviews

A total of 31 semi-structured interviews were conducted in *Lao Theung* language with onsite translation by a bilingual volunteer or a local coordinator after the MDA rounds (in May, June and July, 2016). Respondents were drawn from the two intervention villages, with 16 participants from PMM and 15 from TT. In meetings after the first round of MDA, community members were asked if they were interested in participating in an SSI. Interested community members were visited at their household for interview by a social scientist. Interviews with the same participants were conducted after each round of MDA. During the SSIs, perceptions and experiences of the TME study were explored. All respondents gave written informed consent. The SSIs after round one were recorded using an audio-recording device and notes were taken.

### Data analysis

Audio-recorded data from FGDs and SSIs were transcribed verbatim and translated into English. Together with the field notes, all the data underwent qualitative content analysis (QSR NVivo 11). The codebook developed for initial analysis was based on the research questions (deductive approach) and supplemented by codes that emerged (inductive approach) during the process of reading and coding the transcripts. The transcripts and the field notes were coded line-by-line and resulting codes were analyzed for patterns and relationship to the research question. The content of the codes guided the themes that are presented in the results.

## Results

### Characteristics of participants

FGDs: a total of 100 respondents in four villages (52 from intervention and 48 from control villages) participated in six FGDs conducted for female and six for male participants (Table 1). A minimum of eight and maximum of ten community members participated in each FGD. Overall 52 men (28 from intervention and 24 from control villages) and 48 women (24 from intervention and 24 from control villages) participated in the FGDs. The mean age of the participants were 37 years (range 18–80 years) and all were farmers. The majority of the participants (82/100) reported no formal school education.

SSI: thirty-one participants (15 from PMM and 16 from TT) took part in semi-structured interviews from two of the intervention villages and the same respondents were followed after each rounds of MDA for total of 3 rounds (Table 2). The mean age of the SSI participants was 37 years (range: 18–70 years) and 61% of participants were male (19/31). The majority (28/31; 90.3%) were farmers and (19/31; 61.3%) did not attend school.

**Table 2:** Socio-demographic characteristics of SSI participants (n = 31)

Characteristics	Total	Villages^a^	
		PMM (n = 15)	TT (n = 16)
	Number (%)	Number (%)	Number (%)
Age group (years)			
18–30	15 (48.4)	7 (46.7)	8 (50)
31–40	6 (19.4)	3 (20)	3 (18.8)
≥ 41	10 (32.3)	5 (33.3)	5 (31.3)
Mean = 37.35 ± 16.23; range = 18–70 years			
Sex			
Female	12 (38.7)	6 (40)	6 (37.5)
Male	19 (61.3)	9 (60)	10 (62.5)
Occupation			
Farmer	28 (90.3)	14 (93.3)	14 (87.5)
Teacher	2 (6.5)	1 (6.7)	1 (6.3)
Trader	1 (3.2)	0	1 (6.3)
Education groups			
Not attended school	19 (61.3)	10 (66.7)	9 (56.3)
1–5 years	9 (29)	4 (26.7)	5 (31.3)
>6 years	3 (9.7)	1 (6.7)	2 (12.5)

^a^SSI participants were from two intervention villages

### Why did community members participate in targeted malaria elimination?

**Fig. 5:**
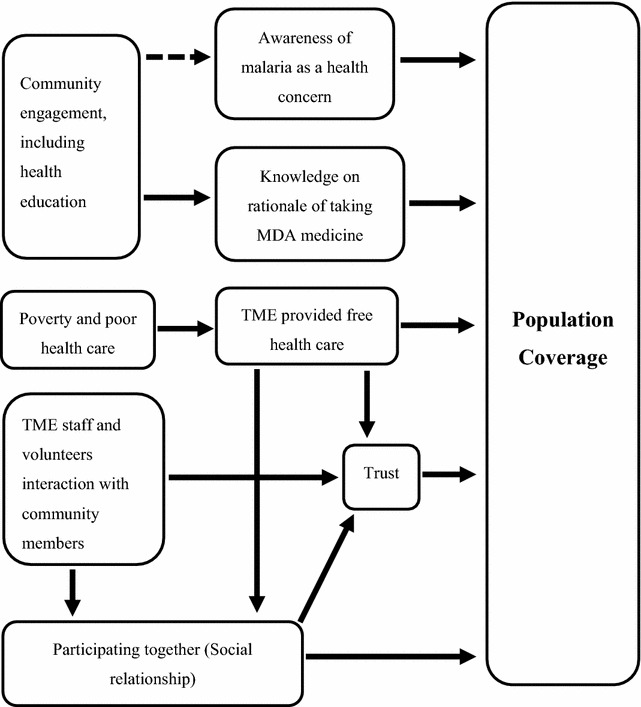
Factors affecting population coverage and participation

Participation in MDA as part of TME in Laos was influenced by diverse contextual and study-specific factors (Fig. 5). These included the initial recognition of malaria as a health concern and the subsequent increase in familiarity with the rationale for MDA. Providing free health care as part of the study was highly valued because—under normal circumstances—access to healthcare was complicated by poverty and distance to the nearest facilities. The partnership that study staff developed with community members to design and implement TME, plus the collaboration with provincial, district, village and the health centre authority figures contributed to building trust in TME. A tendency toward social conformity also played a role in prompting participation.

### Malaria as a health concern

In all four villages, malaria was recognized as a major health problem. In the *Lao Theung* language, malaria was translated as “disease-mosquito bite/*aai moi kap*” and “disease-fever with chills/*aai singyet*”. Most respondents described the prominent symptoms of malaria and its likelihood of causing severe illness and death. Respondents were also familiar with protective measures, such as wearing long sleeved clothes and sleeping under mosquito nets. In the initial FGDs, the participants described malaria in terms of a symptomatic illness. They also linked malaria with visiting the forest or rice fields and poor hygiene. Respondents were open to the idea of asymptomatic malaria among the residents of their villages, in contrast to outsiders (such as the TME staff) who they viewed as cleaner or more hygienic, and who did not go to the forest and rice fields. Their understanding of asymptomatic malaria was therefore associative (to the life style and occupation) and did not include reference to parasite loads in blood or its propensity to cause various types of infection.

*I: Do you think that a healthy person can have malaria parasite in his/her body? For example: me, do you think I have malaria parasite in my body or not?*

*R: No, because you look clean. You do not have to go to forest like us. For us, we have to go to forest every day to find food. We do not have good clothes. I just only have one blanket in my house, I have to give it to my husband and my kids.*

30 year female FGD participant from sub-village of TT with total of eight participants.

*I: Can you tell me about malaria symptoms?*

*R: the symptom of malaria is shivering…… then we have to keep (ourselves) warm, nausea and thirst.*

*I: How do you know that you have malaria?*

*R: when we get [malaria] we will feel shivering, headache, pain abdomen and have Diarrhea.*

*I: Do you go to check at the health center?*

*R: Yes!*

*I: After (the) check, what did a doctor do?*

*R: The doctor gave us a report and gave medicine to us.*

SSI1 P9, 32 year, male, farmer, PMM village

Other than referring to a malaria as a disease, no respondents spontaneously mentioned species (such as falciparum and vivax) of malaria.

#### Poverty and demand for health care

In the TME villages, poverty affected all aspects of daily life. Access to healthcare in general was impeded by difficulties in meeting travel and opportunity (time) costs, and paying up-front treatment charges.

*I: Are there reasons why someone in this village would not seek treatment?*

*R2: No money*

*R7: No transportation*

*I2: …What about other reasons why someone in this village would not seek treatment?*

*R7: The road is not comfortable: especially in the rainy season, it is very difficult to go anywhere.*

*I1: Anything else? Please try to think.*

*R7: If there is nobody to take care of one’s house, find food and take care of one’s children.*

*R8: No driver.*

FGD with eight women at PMM village

Issues of access to health care were prominent in the FGDs and apparent during observations during initial visits to the villages, 5 months before MDA. For example, study staff saw overwhelming demand at the mobile health facility, which was provided by TME staff at community engagement events. This highlight an unmet demand for health care and prompted TME staff to offer free and accessible health care during the study.

Intravenous (IV) drips were in particular demand among residents of the target villages. This demand was accentuated in intervention villages after the first round of MDA, when community members reported adverse events. Community members viewed IV infusions as a quick way to replenish blood and energy. The IV fluids were acquired from health facilities and usually contained a combination of saline solution and vitamins or sometimes additional intramuscular steroids. During severe bouts of illness, such as fever and dehydration due to acute diarrhea, community members often resorted to IV infusions. During informal conversations, respondents reported IV infusions leading to a quick recovery and described a feeling of fluid having been added to the body. In light of their popularity, health staff charged relatively high prices for IV infusions. During TME, the demand for IV infusions from the study physicians (who were offering treatment free of charge) was such that one physician requested a village meeting to explain the appropriate use of IV infusions and their dangers.

### Understandings of mass anti-malarial administration

After each round of MDA, SSI respondents were asked about the rationale for taking medicine while asymptomatic. The majority gave reasons such as “to kill the malaria parasite in their body” and they “wanted to be healthy”. Respondents were positive about the idea of MDA even though sometimes they did not fully understand the rationale behind this approach or expect any specific health benefit. Nonetheless, participation was often motivated by a desire to improve one’s health. This was accentuated by pre-existing health conditions, and overall poor health. Such reports were common among participants irrespective of gender and age.

*I: what do you think about taking medicine, if you are not sick?*

*R: I do not think about anything, I take it because I want to be healthy.*

SSI1P6, 28 year, female, farmer, PMM village

*I: What did you think about taking medicine if you are not sick?*

*R: I am very happy to take medicine but I don’t like the tiredness and headache.*

SSI P15, 25 years, female, farmer, PMM village

Almost all respondents described the TME study in terms of malaria, for example, describing the aim as “to eliminate malaria”, “to treat malaria” or “to help us away from malaria”. These responses reflected the way in which TME was presented to the communities: during meetings with community members, staff reiterated the goal of eliminating malaria and the appeal to work together to achieve the better health. Study posters and other material emphasized this message.

*I: Do you know what is this project doing here in your village?*

*R: Malaria treatment to eliminate malaria away from our village.*

*I: What is the aim of the project?*

*R: To make us having a good health.*

SSI P10, 40 years, female, farmer, PMM village

Assessing attitudes towards the community engagement activities that accompanied the clinical activities was complicated by a tendency to view all TME activities as a single undertaking. Other than remembering study activities, such as video shows, meetings, home visits, blood test and taking medicine, community members did not talk spontaneously about their preference for particular activities. Upon further probing, respondents often expressed that “they liked [it] all”. Participants often said they received the information on TME either from volunteers or village head, mostly during the meetings and night video shows. Often during informal conversations with community members, concerning their reasons for participation, they mentioned “to improve one’s health” and “to get rid of malaria”.

*I: How did you learn about the project?*

*R: I first heard from village head and volunteers and also during meetings when they explained poster.*

*I: Did you join any other activities or meeting of our project?*

*R: Yes! When your project come to do night video shows and during MDA.*

*I: Did you explain about our project to another person?*

*R: Yes! I talked to villagers that this project is helping poor people by giving them malaria medicine.*

SSI1 P31, 34 years, female, teacher, TT main sub-village

### Reported adverse events and other concerns about the anti-malarials

After the first round of MDA, many community members reported adverse events, particularly malaise or tiredness *(Lao Theung: Yï*-*yo)*, decreased appetite (*Lao Theung: Tà é chadoí*), sense of fullness of abdomen (*Lao Theung: pasai chà)* and food does not taste good (*Lao Theung: chadoí ta ém*), headache (*Lao Theung: Tï Plä*) and sleepiness (*Lao Theung: Yì Bè*). These events prompted participants to visit local health facilities and request restorative treatment in the form of an IV infusion. In addition to saline solution and vitamins, they requested IV steroids often referred as “*Kakok*”.

In response to these reports, the study team augmented the health care provision: study physicians subsequently spent more time in the villages; TME staff liaised with the health centre staff; the quantity and range of medicines available free of charge was increased; and health centre staff along with TME staff visited the households of participants who reported complaints. Meetings were held to explain and answer the adverse events such as tiredness, loss of appetite and epigastric pain. Apart from providing the symptom-specific treatment, TME staff offered counseling. Specific messages from study staff included:

*Any medicine can cause certain discomfort*

*New medicines are generally less tolerated at first*

*The medicine is fighting against the parasite in your body*

*Please feel free to consult our doctors if you have such problems*

During the meetings, the specific risks and benefits associated with the IV medicines were discussed.

Soon after increasing health care and education, observations indicated a decline in the number of participants attending health centres with complaints regarding adverse events. In spite of the adverse events, members of both intervention villages participated in subsequent rounds of MDA. Thus the population coverage was stable in both intervention villages in all three rounds (in PMM, coverage was 87, 86 and 87% in three respective rounds and in TT, coverage was 87, 85 and 88% in three rounds respectively).

*I: What was the nature of tiredness, to what extent were you able to work?*

*R: I could walk, go to the toilet, work at home but could not go to the field.*

*I: What was the nature of “loss of appetite”?*

*R: It was decrease in appetite but not complete decrease. The food doesn’t taste delicious… and we feel that medicine is still inside the stomach.*

Informal conversation with a resident of PMM after TME round 1

Community members raised concerns about the blood collection (up to 3 ml) following TME: they suggested that “blood is forcefully drawn”, that “large quantity of blood is drawn” or they reported being “afraid of needles”. These stories had an impact on recruitment, with some who refused to participate citing blood sampling, and specifically the large quantity of blood drawn, as reasons for their reluctance to participate. Such groups of refusers were often clusters of friends of relatives. This clustering of refusers was also indicative of a more general trend in behavior. In informal conversations, locals described how, “if all participate, I will participate”.

The stories emerged in intervention villages after the first round of MDA and spread quickly to neighboring control villages. Aware of this, study staff responded by involving relevant village authority figures (for example, the village head) in community meetings. The village head from TT (residents of this village were said to be responsible for spreading the rumour) explained and answered questions, with the support of the TME team. The following day, the majority of community members participated in the baseline survey (coverage was 90% in XT).

### Participating together

Study staff viewed frequent social visits and participating in village activities as a high priority. These included playing pétanque, volleyball, football and *sepák tákraw* ( ) with community members. Staff also participated in cultural gatherings, such as *Champhi* (religious sanctification ceremony to cleanse a bad spirit that causes diseases to a family member), and funeral ceremonies. During these events, staff ate and drank (often local alcohol [*Lao Khao*]) with community members. This helped to build rapport. Frequent visits to the villages, overnight stays, and the fact that TME physicians were resident in local health centers strengthened relationships between study staff and community members. Sometimes, participants requested a home visit to see their family members who had been too sick to come to the health center. During home visits, study staff offered diagnosis and treatment whenever possible or help patients to be transported to a referral hospital. Such interactions helped study staff to better understand the nuances and subtle reactions of participants’ health care requirements and the need to adapt TME activities. Study staff scheduled study activities so as to respect work routines and festivities. This included re-opening the MDA venues in sub-villages, adapting the timing of household visits to offer medicine and health care.

## Discussion

The Laos TME project achieved an 87% population coverage for all three rounds potentially sufficient to interrupt malaria transmission. With a background of a community engagement that entailed partnering the research with local volunteers and the authorities, provision of free health care and incentives, various factors contributed to this achievement: respondents’ perceptions of malaria and their health care needs; experiences of adverse events, the responses of study staff; and the social relationships that staff developed with participants.

### Malaria as a health concern

As was recently found in Cambodia, respondents in Laos were familiar with malaria as a symptomatic illness of malaria and recognized it as an important health concern. In both sites, despite gaps in awareness of the details of malaria infection, respondents expressed positive opinions about malaria elimination efforts [11]. As has been described elsewhere in Asia and Africa [27], respondents linked malaria infection to unhygienic conditions, particularly poor water and sanitation [28, 29]. The associations that respondents made between “being unclean”, “visiting local forests” or their “rice fields” and malaria infection meant that they were receptive to the concept of asymptomatic malaria among the residents of their villages and the need to clear latent infections. Although asymptomatic malaria was not perceived to be linked to parasite loads or its propensity to cause various types of infection, such an understanding seemingly facilitated the acceptance of taking an anti-malarial when not ill.

In Cambodia and Laos, respondents were largely unaware of the different malaria species present locally (*Plasmodium vivax* and *Plasmodium falciparum*) [11]. This is unsurprising because the health education that accompanied TME did not deal with such intricacies [11, 20]. Future malaria elimination campaigns will need to address the importance of vivax malaria which are recognized to be the long-term reservoir for sub-clinical malaria infection [30, 31] and require a treatment regimen including 8-aminoquinolines, such as primaquine [32].

### Poverty and health care

Community members were engaged in subsistence farming and had very little access to cash. The resulting poverty restricted their access to health care. Offering a monetary incentive (USD ~ 12 per round that entailed 3 days participation) and providing free health care encouraged community members to participate. This was particularly important in terms of palliating the opportunity costs that missing a day’s labour in the field or forest entailed [20]. The formative research was key to designing the nature of the incentives, particularly how the health care was offered [20].

Clinical trials, particularly in low and middle-income countries, where health care infrastructure is weak or difficult to access, often offer ancillary care to participants [33–38]. In Malawi, the provision of ancillary care for clinical trial participants was considered ethically imperative [39]. Ancillary care has also been integrated into the clinical trials run by Shoklo Malaria Research Unit (SMRU), which often recruit vulnerable populations on the Thai-Myanmar border [37].

Many ethicists view the provision of ancillary care as an essential component of ethical research, which must be delineated beforehand and take into account participants’ needs, the capacity to help and the investigators’ level of engagement with participants [33–37]. Ancillary care within Laos TME included treating a wide range of diseases and was responsive to local health care needs. The approach followed reflects that of SMRU [37] and is recommended by some ethicists [34, 36, 40].

The ancillary care often has additional symbolic value for study participants: it indicates an attentiveness to their suffering, engenders a sense of a supportive social relationship and generates trust in the research team. The experiences of participants in Laos TME, echoed those clinical trial participants in The Gambia, for whom the free study health care became conflated with social relationships with study staff, generating feelings of trust, reciprocity and interdependence [41].

### Understandings of mass anti-malarial administration

As part of community engagement for TME, staff used multiple health education methods to explain the concept of asymptomatic malaria, the rationale for blood testing and MDA. In addition, a community-wide appeal to eliminate malaria from the village was made through posters in village meetings [20, 22]. Participants’ responses indicate that these efforts had some impact, particularly through their familiarity with the overall aim of the study. Their impact on awareness of the details of asymptomatic malaria and the study rationale was less clear. A questionnaire-based survey that accompanied this research also highlighted how familiarity with the rationale for blood testing and MDA contributed to complete participation in Laos TME [22]. In The Gambia, understanding the rationale of MDA was found to contribute to the participation [42], and in recent MDAs conducted in Vietnam [13] and Thai-Myanmar border [9] inadequate understanding of the rationale of MDA was associated with non-participation.

Familiarity with the study rationale and knowledge of malaria does not always translate into participation in MDA. For example, in one community on the Thai-Myanmar border, although participants demonstrated an increase in their knowledge of malaria over the study period, participation in the MDA remained low. Study staff attributed the low participation to political divisions and the antagonism between fractions of the community [9]. This highlights the limitation of health education. It also demonstrates the importance of understanding the local social and cultural context, and incorporating these insights into the design of MDA and the accompanying community engagement. In the Lao TME project, study staff quickly recognized a preference for dedicated, exclusive study activities in the sub-villages and adapted their approach accordingly [20].

### Reported adverse events and intravenous fluid

Real and perceived adverse events after the first round of MDA prompted many community members to seek IV medication. This was rooted in the belief that IV infusions replenish blood and provide energy. Studies of demand for IV medication have highlighted similar perceptions, in north-eastern Thailand (bordering Laos) [43], Indonesia and Uganda [44] and amongst both clinicians and patients [45].

Participants in TME in Cambodia also sought IV medication without clinical justification [11]. This presents a challenge for clinical trials in the region that provide ancillary care. In Lao TME, a multi-pronged approach was taken to counteracting the demand for IV infusions. This included providing health education during community meetings and home visits and intensifying the ancillary health care. This responsive approach was an essential element of the community engagement and contributed to maintaining high population coverage in subsequent rounds of MDA [20]. Similarly, in Vanuatu [46] and Cambodia, [11], the capacity to respond to participants’ concerns was key to ensuring high participation throughout multiple rounds of MDA.

### Participating together and trust

In The Gambia, study staff developed social bonds with trial participants that went beyond the formal researcher-subject relationship, and fostered participation [41]. Similarly, in Myanmar, TME study staff’s involvement in village life—sharing food with community members and participating in local social activities, such as, funerals and festivals—contributed to the uptake of MDA [10]. In the TME villages, study staff observed a general conformism, with individual’s behavior often influenced by that of other household members and neighbours. As has been generally described in *Lao Theung* society, there was also interdependence between households, reflected in sharing labour, communal problem solving and decision-making [22, 47]. This affected participation in TME and often during informal/formal conversations, respondents described how, “if all participate, I will participate”. Notably, on the Thai-Myanmar border, MDA coverage was much higher in cohesive than in politically fragmented communities [9]. The questionnaire-based study and quantitative analysis of factors affecting participation in TME also highlighted the influence of other household members on uptake of MDA [22]. This reflects the high value placed on familial conformity in Laos [21, 48].

### Strengths and limitations

This study used multiple methods at various time points (FGDs at baseline and SSIs after MDA), supplemented by observations throughout the study period (Fig. 2). Three researchers collected data to reduce the possibility of bias caused by the influence of a single data collector. Cultural modesty and conformism might have affected the data from FGDs and SSIs. However, participants were vocal about the adverse events and in their demand for additional health care. In addition, the TME team’s prolonged deployment in the villages, which included overnight stays, helped to develop rapport and to understand the nuances and subtleties of participants’ reactions.

The findings are limited by the fact that this study was conducted in the context of clinical trial with meticulous procedures along with participant incentives (cash, free health care, T-shirts, sweets, household utensils), and improvement to water and sanitation through installation of water pumps. Control programmes with limited resources for incentives and investment in health care and improvement in water and sanitation may not be able to offer similar benefits. Also, MDAs for disease control and elimination in non-clinical trial context which may not require frequent blood sampling might be able to achieve higher population coverage. This study derived the data from on-site translation from bilingual translators who were fluent in *Lao* and *Lao Theung*. As *Lao Theung* language is an oral language without a written script, the nuances and meaning could have been lost during translation.

## Conclusion

In the target communities in Nong District, the coverage of MDA (and blood survey) was high. Several factors contributed to this: participants’ openness to the concept of asymptomatic malaria and their awareness of the disease as a health problem and of the study aim; providing health care—as part of TME—to communities that have limited access to medical services; a responsive approach to community engagement, whereby concerns were addressed promptly; and the social bonds between staff and community members that promoted trust in the study procedures. Investing in community engagement that builds partnerships with local volunteers and authorities, responds to their concerns and is adapted to the local context is likely to improve effectiveness of future malaria control programmes, including those involving MDA. Further research in circumstances that more closely approximate implementation is needed to better inform the design of MDA within malaria control programs in the region.

## Additional files

**Additional file 1.** Community engagement activities in Laos TME.

**Additional file 2.** Malaria guideline book for community members (in English).

## References

[1] Ashley EA, Dhorda M, Fairhurst RM, Amaratunga C, Lim P, Suon S (2014). Spread of artemisinin resistance in *Plasmodium falciparum* malaria. N Engl J Med.

[2] Mbengue A, Bhattacharjee S, Pandharkar T, Liu H, Estiu G, Stahelin RV (2015). A molecular mechanism of artemisinin resistance in *Plasmodium falciparum* malaria. Nature.

[3] WHO. Accelerating malaria elimination in the Greater Mekong Subregion. Geneva: World Health Organization; 2014. http://www.who.int/malaria/areas/greater_mekong/overview/en/. Accessed 13 July 2017.

[4] WHO. Strategy for Malaria Elimination in the Greater Mekong Subregion (2015–2030). Geneva: World Health Organization; 2015. http://iris.wpro.who.int/bitstream/handle/10665.1/10945/9789290617181_eng.pdf?sequence=1. Accessed 14 July 2017.

[5] Imwong M, Suwannasin K, Kunasol C, Sutawong K, Mayxay M, Rekol H (2017). The spread of artemisinin-resistant *Plasmodium falciparum* in the Greater Mekong subregion: a molecular epidemiology observational study. Lancet Infect Dis.

[6] von Seidlein L, Dondorp A (2015). Fighting fire with fire: mass antimalarial drug administrations in an era of antimalarial resistance. Expert Rev Anti Infect Ther.

[7] Hanboonkunupakarn B, White NJ (2016). The threat of artemisinin resistant malaria in Southeast Asia. Travel Med Infect Dis.

[8] Lwin KM, Imwong M, Suangkanarat P, Jeeyapant A, Vihokhern B, Wongsaen K (2015). Elimination of *Plasmodium falciparum* in an area of multi-drug resistance. Malar J.

[9] Kajeechiwa L, Thwin MM, Shee PW, Yee NL, Elvina E, Peapah P (2016). The acceptability of mass administrations of anti-malarial drugs as part of targeted malaria elimination in villages along the Thai-Myanmar border. Malar J.

[10] Sahan K, Pell C, Smithuis F, Phyo AK, Maung SM, Indrasuta C (2017). Community engagement and the social context of targeted malaria treatment: a qualitative study in Kayin (Karen) State, Myanmar. Malar J.

[11] Pell C, Tripura R, Nguon C, Cheah P, Davoeung C, Heng C (2017). Mass anti-malarial administration in western Cambodia: a qualitative study of factors affecting coverage. Malar J.

[12] Tripura R, Peto TJ, Veugen CC, Nguon C, Davoeung C, James N (2017). Submicroscopic Plasmodium prevalence in relation to malaria incidence in 20 villages in western Cambodia. Malar J.

[13] Nguyen TN, Thu PN, Hung NT, Son DH, Tien NT, Van Dung N (2017). Community perceptions of targeted anti-malarial mass drug administrations in two provinces in Vietnam: a quantitative survey. Malar J.

[14] Phommasone K, Adhikari B, Henriques G, Pongvongsa T, Phongmany P, von Seidlein L (2016). Asymptomatic Plasmodium infections in 18 villages of southern Savannakhet Province, Lao PDR (Laos). Malar J.

[15] Adhikari B, James N, Newby G, von Seidlein L, White NJ, Day NP (2016). Community engagement and population coverage in mass anti-malarial administrations: a systematic literature review. Malar J.

[16] Newby G, Hwang J, Koita K, Chen I, Greenwood B, von Seidlein L (2015). Review of mass drug administration for malaria and its operational challenges. Am J Trop Med Hyg.

[17] Cheah PY, White NJ (2016). Antimalarial mass drug administration: ethical considerations. Int Health.

[18] Andriesse E, Phommalath A (2013). Provincial poverty dynamics in Lao PDR: a case study of Savannakhet. J Curr Southeast Asian Aff.

[19] International Monetary Fund. National Growth and Poverty Eradication Strategy. International Monetary Fund Publication Services; 2004. https://www.imf.org/external/pubs/ft/scr/2004/cr04393.pdf. Accessed Aug 2017.

[20] Adhikari B, Pell C, Phommasone K, Soundala X, Kommarasy P, Pongvongsa T (2017). Elements of effective community engagement: lessons from a targeted malaria elimination study in Lao PDR (Laos). Glob Health Action.

[21] Ovesen J (2002). Indigenous peoples and development in Laos: ideologies and ironies. Recherche en sciences humaines sur l’Asie du Sud-Est.

[22] Adhikari B, Phommasone K, Pongvongsa T, Kommarasy P, Soundala X, Henriques G (2017). Factors associated with population coverage of targeted malaria elimination (TME) in southern Savannakhet Province, Lao PDR. Malar J.

[23] von Seidlein L. MORU TME brings water pumps to Laos villages; 2017. http://www.tropmedres.ac/moru-tme-brings-water-pumps-to-laos-villages. Accessed Aug 2017.

[24] Adhikari B, Mishra SR, Raut S (2016). Rebuilding earthquake struck Nepal through community engagement. Front Public Health.

[25] Clinical and Translational Science Awards (CTSA) (2011). Principles of community engagement. NIH Publication No 11-7782.

[26] Tindana PO, Singh JA, Tracy CS, Upshur RE, Daar AS, Singer PA (2007). Grand challenges in global health: community engagement in research in developing countries. PLoS Med.

[27] Pell C, Straus L, Andrew EV, Menaca A, Pool R (2011). Social and cultural factors affecting uptake of interventions for malaria in pregnancy in Africa: a systematic review of the qualitative research. PLoS ONE.

[28] Andrew EV, Pell C, Angwin A, Auwun A, Daniels J, Mueller I (2015). Knowledge, attitudes, and practices concerning malaria in pregnancy: results from a qualitative study in Madang, Papua New Guinea. PLoS ONE.

[29] Menaca A, Pell C, Manda-Taylor L, Chatio S, Afrah NA, Were F (2013). Local illness concepts and their relevance for the prevention and control of malaria during pregnancy in Ghana, Kenya and Malawi: findings from a comparative qualitative study. Malar J.

[30] Peto TJ, Kloprogge SE, Tripura R, Nguon C, Sanann N, Yok S (2016). History of malaria treatment as a predictor of subsequent subclinical parasitaemia: a cross-sectional survey and malaria case records from three villages in Pailin, western Cambodia. Malar J.

[31] Tripura R, Peto TJ, Chalk J, Lee SJ, Sirithiranont P, Nguon C (2016). Persistent *Plasmodium falciparum* and *Plasmodium vivax* infections in a western Cambodian population: implications for prevention, treatment and elimination strategies. Malar J.

[32] Chu CS, White NJ (2016). Management of relapsing *Plasmodium vivax* malaria. Expert Rev Anti Infect Ther.

[33] Haire BG, Ogundokun O (2014). Ethics of ancillary care in clinical trials in low income countries: a Nigerian case study. Afr J Reprod Health.

[34] Participants in Georgetown University Workshop on Ancillary-care obligations of medical researchers working in developing countries. The ancillary-care obligations of medical researchers working in developing countries. PLoS Med. 2008; 5:e90. 10.1371/journal.pmed.0050090 PMC2386835 18494553

[35] Belsky L, Richardson HS (2004). Medical researchers’ ancillary clinical care responsibilities. BMJ.

[36] Dickert N, DeRiemer K, Duffy PE, Garcia-Garcia L, Mutabingwa TK, Sina BJ (2007). Ancillary-care responsibilities in observational research: two cases, two issues. Lancet.

[37] Pratt B, Zion D, Lwin KM, Cheah PY, Nosten F, Loff B (2013). Ancillary care: from theory to practice in international clinical research. Public Health Ethics.

[38] Molyneux S, Mulupi S, Mbaabu L, Marsh V (2012). Benefits and payments for research participants: experiences and views from a research centre on the Kenyan coast. BMC Med Ethics.

[39] Mfutso-Bengo J, Ndebele P, Jumbe V, Mkunthi M, Masiye F, Molyneux S (2008). Why do individuals agree to enrol in clinical trials? A qualitative study of health research participation in Blantyre, Malawi. Malawi Med J.

[40] Dickert N, Wendler D (2009). Ancillary care obligations of medical researchers. JAMA.

[41] Geissler PW, Kelly A, Imoukhuede B, Pool R (2008). He is now like a brother, I can even give him some blood’–relational ethics and material exchanges in a malaria vaccine ‘trial community, The Gambia. Soc Sci Med.

[42] De Martin S, von Seidlein L, Deen JL, Pinder M, Walraven G, Greenwood B (2001). Community perceptions of a mass administration of an antimalarial drug combination in The Gambia. Trop Med Int Health.

[43] Reeler AV (1990). Injections: a fatal attraction?. Soc Sci Med.

[44] Van Staa A, Hardon A. Injection practices in the developing world. Geneva: World Health Organization, WHO/DAP/964; 1996.

[45] Li HK, Agweyu A, English M, Bejon P (2015). An unsupported preference for intravenous antibiotics. PLoS Med.

[46] Kaneko A (2010). A community-directed strategy for sustainable malaria elimination on islands: short-term MDA integrated with ITNs and robust surveillance. Acta Trop.

[47] Trankell IB (1999). On the road in Laos. An anthropological study of road construction and rural communities.

[48] Hockings P (1993). Encyclopedia of World Cultures.

