# Supplementary material for: Why do people participate in mass anti-malarial administration? Findings from a qualitative study in Nong District, Savannakhet Province, Lao PDR (Laos)
Source: Malar J. 2018 Jan 9;17:15. doi: 10.1186/s12936-017-2158-4 (PMC5761145; doi:10.1186/s12936-017-2158-4)
Supplement: Supplementary file 1 — Additional file 1. Community engagement activities in Laos TME. [file 12936_2017_2158_MOESM1_ESM.pdf]

# Selected community engagement activities for Lao TME

| **Table 1: Community Engagement Activities before MDA started** | | | | | | |
|---|---|---|---|---|---|---|
| **Activities** | **Date** | **Other sites** | **Intervention Village** | | **Control Village** | |
| | | | PMM | TT | OTP | XT |
| Authority Sensitization | November $2^{nd}$, 2015 | Provincial Malaria Office, Savannakhet | | | | |
| TME Meeting | November $3^{rd}$, 2015 | LOMWRU Office, Vientiane | | | | |
| CE Multi-site Meeting | November 6-7, 2015 | Community Engagement workshop at MORU | | | | |
| CE book preparation for villagers | November 9-14, 2015 | LOMWRU office, Vientiane | | | | |
| District Level Meeting (A general description of TME as a booklet was distributed) | November 23, 2015 | Nong District Health Office | | | | |
| Baseline survey, Census and GPS | November 25 to December 18, 2015 | | | | | |
| FGT (Focused Group Training) | December 22, 2015 | | | | | |
| | December 23, 2015 | | | | | |
| Design of Poster for CE | December 24, 2015 | At TME office, Nong | | | | |
| Design of Poster for CE | December 25, 2015 | At TME office, Nong | | | | |
| Local CE staffs interview to design the CE material | December 29, 2015 | | | | | |
| | December 30, 2015 | | | | | |
| | December 31, 2015 | | | | | |
| | January $3^{rd}$, 2016 | | | | | |
| Meeting on planning of CE. | January $4^{th}$, 2016 | At Nong District Office | | | | |

CE=Community Engagement; LOMWRU=Lao-Oxford-Mahosot Hospital Wellcome Trust Research Unit; MORU=Mahidol-Oxford Tropical Medicine Research Unit; TME=Targeted Malaria Elimination

**Table 1: Community Engagement Activities before MDA started**

| Activities | Date | Other sites | Intervention Village | | Control Village | |
|---|---|---|---|---|---|---|
| | | | PMM | TT | OTP | XT |
| Trainer's Training (Malaria Guide Book, Poster, Team games) | January 6th, 2016 | | | | ■ | |
| | January 7th, 2016 | | ■ | | | ■ |
| | January 8th, 2016 | | | ■ | | |
| Authority Meeting | January 13th, 2016 | At Nong Meeting Hall | | | | |
| Mass Meeting | January 14th, 2016 | | ■ | | | ■ |
| Mass Meeting | January 15th, 2016 | | | ■ | ■ | |
| Devolvement of Responsibilities to Volunteers | January 18th, 2016 | | ■ | | | ■ |
| Devolvement of Responsibilities to Volunteers | January 19th, 2016 | | | ■ | ■ | |
| Video Preparation | January 20th to January 27th, 2016 | AT Nong TME Office | | | | |
| Evening CE | January 28th, 2016 | | ■ | | | |
| | January 29th, 2016 | | | | | ■ |
| | January 30th, 2016 | | | | ■ | |
| TT Geng and Main | January 31st, 2016 | | | ■ | | |
| Appok | February 1, 2016 | | | ■ | | |
| Consent and House to house sensitization | February 9, 2016 to February 19, 2016 | | ■ | ■ | ■ | ■ |
| Meetings with villagers for consent after encountering rejecters | February 24, 2016 | | ■ | | | |
| Movie preparation on MDA | February 27 to March 11, 2016 | AT Nong TME Office | | | | |
| Night CE | March 16, 2016 | | | | ■ | |
| | March 17, 2016 | | | ■ | | |
| Mobile health camp | March 18, 2016 | | | | | ■ |
| | March 24, 2016- March 26, 2016 | | | ■ | | |
| | March 27, 2016 | | ■ | | | |
| Bed net and kitchen utensil distribution | March 28, 2016 | | ■ | | | |
| | March 29, 2016 | | | ■ | | ■ |
| | March 30, 2016 | | | | ■ | |
| Night CE | April 5, 2016 | | ■ | | | |

CE=Community Engagement; LOMWRU=Lao-Oxford-Mahosot Hospital Wellcome Trust Research Unit; MORU=Mahidol-Oxford Tropical Medicine Research Unit; TME=Targeted Malaria Elimination

| Table 2: Community Engagement activities during and around MDA rounds | | | | | | |
|---|---|---|---|---|---|---|
| **Activities** | **Date** | **Other sites** | **Intervention Village** | | **Control Village** | |
| | | | PMM | TT | OTP | XT |
| Evening and Night CE during MDA | April 21-April 29, 2016 | | | ■ | | |
| Evening and Night CE during MDA | April 30, 2016 | | ■ | | | |
| House to House visits | May 2nd 2016 | | ■ | | | |
| Evening and Night CE during MDA | May 3rd, 2016 | | ■ | | | |
| Night CE during Blood collection | May 7-8th, 2016 | | | | ■ | |
| Night CE during Blood collection | May 11-12th, 2016 | | | | | ■ |
| Volunteers Meeting, House to House visit and Evening CE for Round 2 | May 22-25, 2016 | | ■ | | | |
| Volunteers Meeting, House to House visit and Evening CE for Round 2 | May 29 to June 1 | | | ■ | | |
| Volunteers Meeting, House to House visit and Evening CE for Round 3 | June 26, 2016 | | ■ | | | |
| | June 30th, 2016 and July 4th, 2016 | | | ■ | | |
| Month 3 Survey | | | | | | |
| Evening CE and Volunteers meeting | July 22, 2016 | | ■ | | | |
| Evening CE and Volunteers meeting | July 25, 2016 | | | | | ■ |
| Evening CE and Volunteers meeting | July 26, 2016 | | | | ■ | |
| Evening CE and Volunteers meeting | July 27, 2016 | | | ■ | | |
| Evening CE and Volunteers meeting | July 30, 2016 | | | ■ | | |
| Night CE | August 1, 2016 | | ■ | | | |

CE=Community Engagement; LOMWRU=Lao-Oxford-Mahosot Hospital Wellcome Trust Research Unit; MORU=Mahidol-Oxford Tropical Medicine Research Unit; TME=Targeted Malaria Elimination

**Table 2: Community Engagement activities during and around MDA rounds**

| **Activities** | **Date** | **Other sites** | **Intervention Village** | | **Control Village** | |
|---|---|---|---|---|---|---|
| | | | PMM | TT | OTP | XT |
| *Volunteers, village head, seniors were asked to conduct meeting and house to house counselling at Appok for participation* | August 2, 2016 | | | | | |
| Night CE | August 4, 2016 | | | | | |
| Night CE/ *Volunteers, village head, seniors were asked to conduct meeting and house to house counselling at PMM for participation* | August 5, 2016 | | | | | |
| Night CE | August 7, 2016 | | | | | |
| Night CE | August 8, 2016 | | | | | |
| *Volunteers, village head, seniors were asked to conduct meeting and house to house counselling at Tantip for participation* | August 9, 2016 | | | | | |

CE=Community Engagement; LOMWRU=Lao-Oxford-Mahosot Hospital Wellcome Trust Research Unit; MORU=Mahidol-Oxford Tropical Medicine Research Unit; TME=Targeted Malaria Elimination
