# Supplementary material for: Why do people participate in mass anti-malarial administration? Findings from a qualitative study in Nong District, Savannakhet Province, Lao PDR (Laos)
Source: Malar J. 2018 Jan 9;17:15. doi: 10.1186/s12936-017-2158-4 (PMC5761145; doi:10.1186/s12936-017-2158-4)

Malaria Guideline Book

wellcome trust
Laos Oxford
LOMWRU

Introduction

1. Who are we?

   We are doctors and social scientists from Mahosot Hospital, Vientiane.

2. What we do?

   We work with the community to find out and eliminate malaria from your village

3. How do we do?

   We will first identify all healthy persons with malaria parasites in their blood and we will treat them all-this is called MDA (Mass Drug Administration)

Asymptomatic Malaria

1. What is asymptomatic malaria?

Asymptomatic malaria is a person who has few malaria parasites in his body, but he looks healthy and can do every thing like a normal person.

2. Why don't they feel sick?

Because they have less malaria parasites in their body.

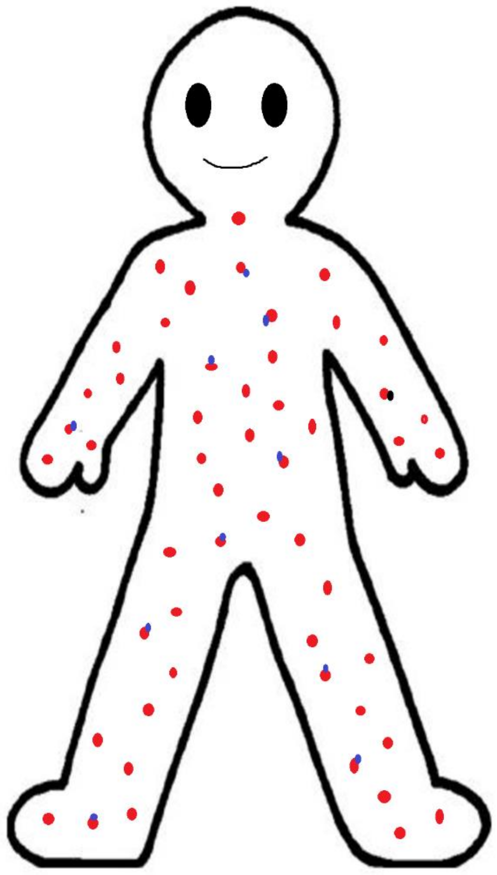

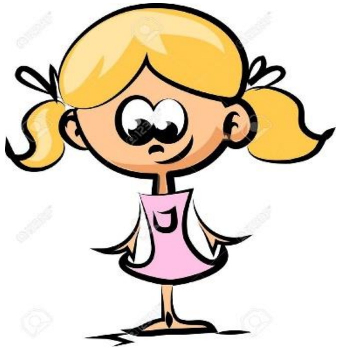

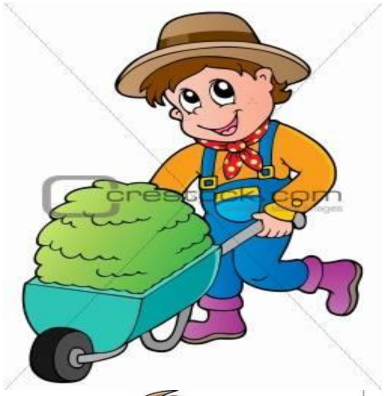

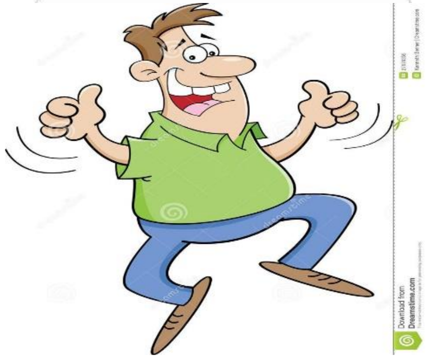

Is the Asymptomatic malaria dangerous?

Yes, it is dangerous because:

1. It can transmit to the other people
2. It can change from asymptomatic malaria to symptomatic malaria
3. It is difficult to detect by normal method
4. You can die because of it

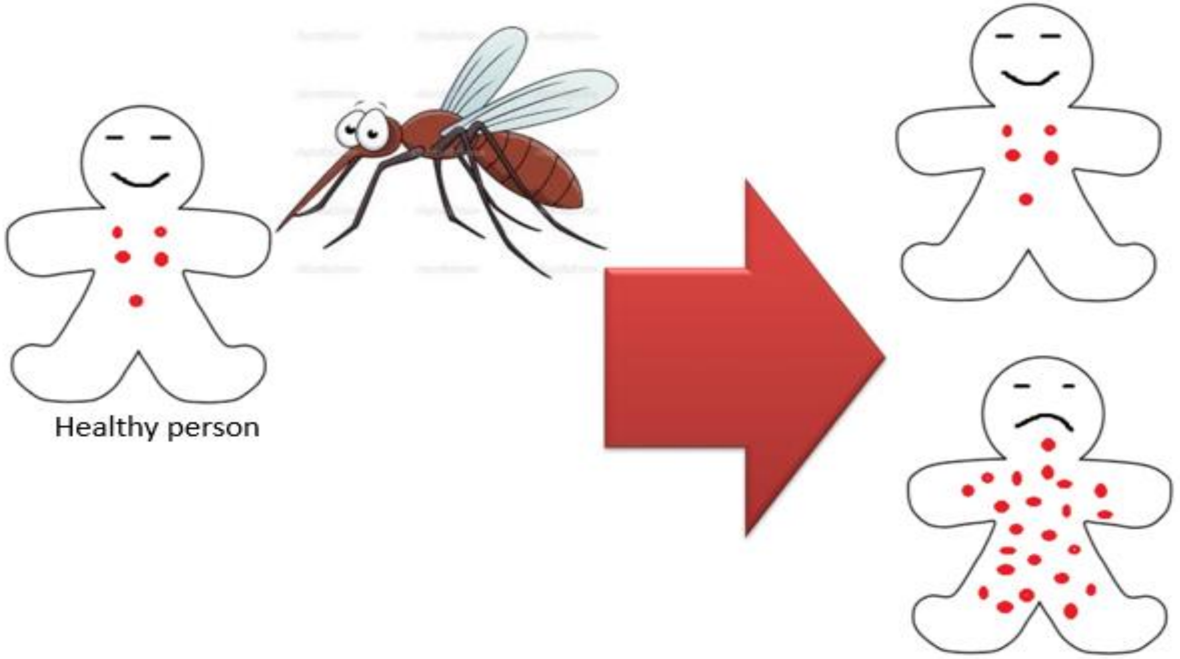

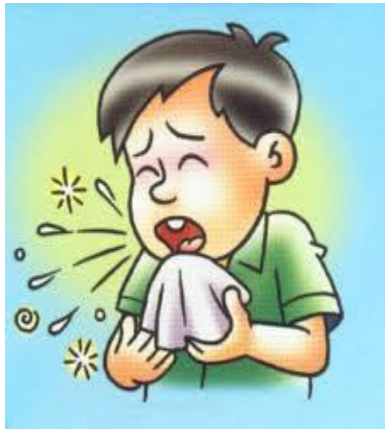

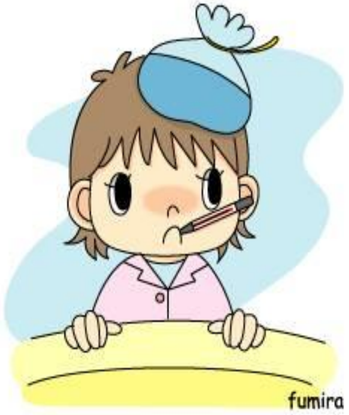

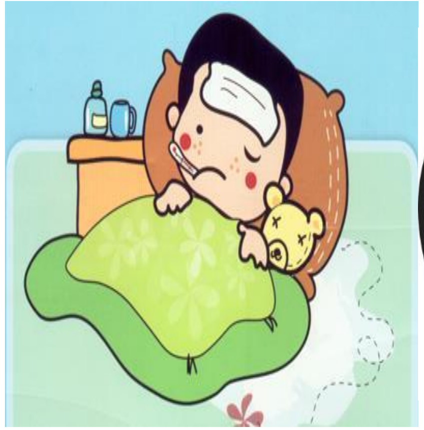

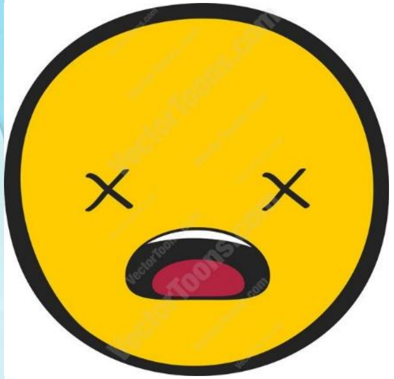

How do we know that we have an asymptomatic malaria?

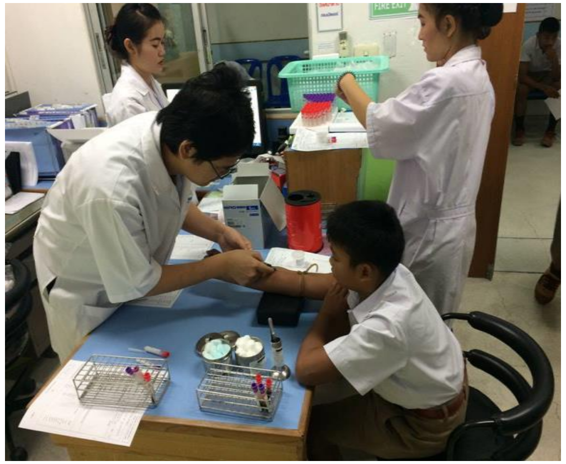

- One way to find out the malaria parasite in our body is by doing the blood test.
- We need more blood to identify the parasite because there are few in number.
- For adult we need 3cc
- For a children under 5 year we need 1cc

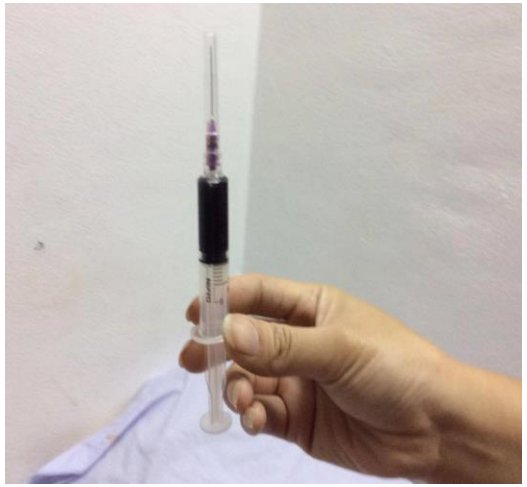

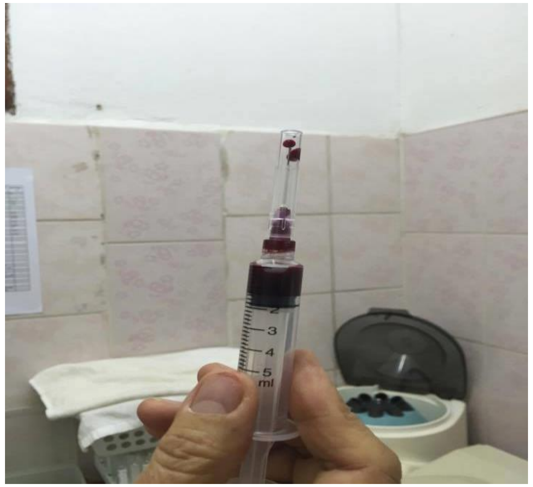

What should we do with asymptomatic malaria?

We should eliminate them because it can be transmitted to other healthy persons

(when the mosquito bites an asymptomatic person and then bites the other people in the village, all of them will get malaria)

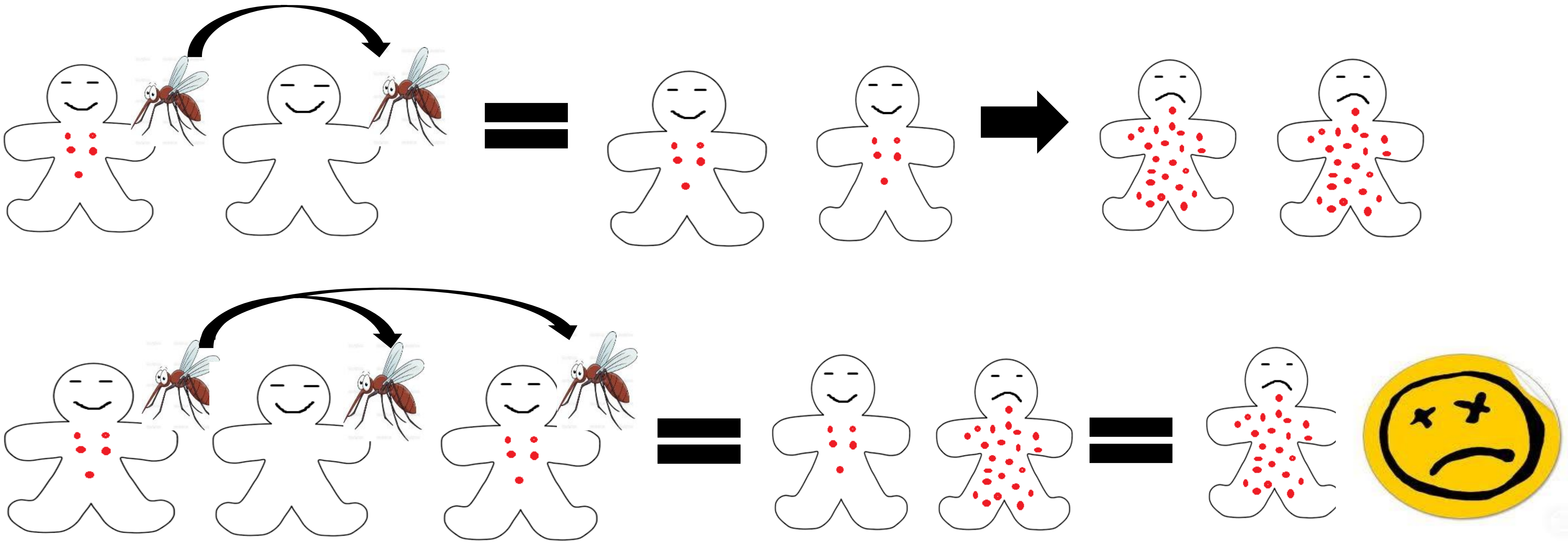

How can we eliminate them

The only way to eliminate malaria is By taking medicine (all the villagers)

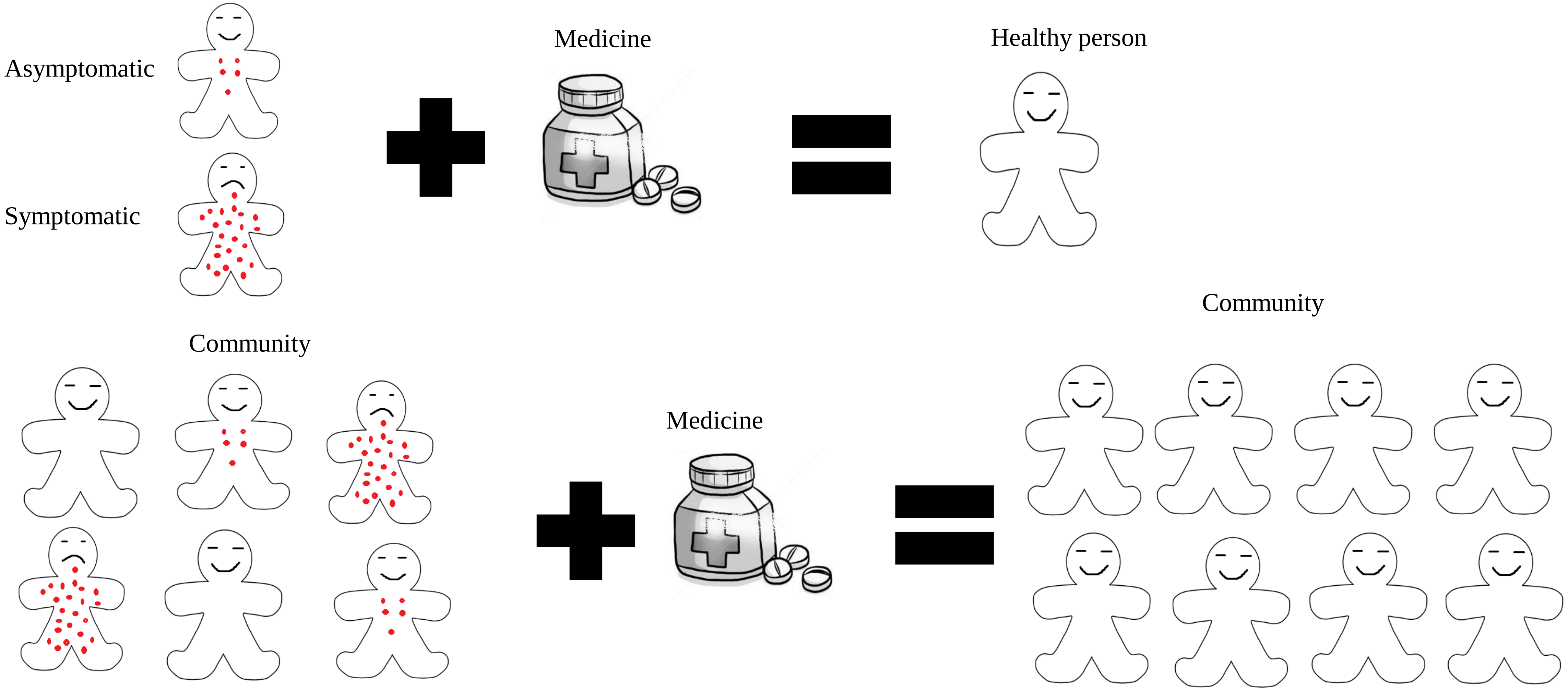

Supplement: Supplementary file 2 — Additional file 2. Malaria guideline book for community members (in English). [file 12936_2017_2158_MOESM2_ESM.pdf]
